# Simvastatin and Bezafibrate ameliorate Emotional disorder Induced by High fat diet in C57BL/6 mice

**DOI:** 10.1038/s41598-017-02576-5

**Published:** 2017-05-24

**Authors:** Hui Wang, Jia Zhou, Qiong Zhen Liu, Lu Lu Wang, Jing Shang

**Affiliations:** 1 State Key Laboratory of Natural Medicines, China Pharmaceutical University, Nanjing, 210009 China; 2 Jiangsu Key Laboratory of TCM Evaluation and Translational Research, China Pharmaceutical University, Nanjing, 211198 China; 3 Qinghai Key Laboratory of Tibetan Medicine Pharmacology and Safety Evaluation, Northwest Institute of Plateau Biology, Chinese Academy of Sciences, Xining, 810008 Qinghai Province P.R. China

## Abstract

High fat diet (HFD)-induced metabolic disorders may lead to emotional disorders. This study aimed to explore the effect of simvastatin (SMV) and bezafibrate (BZ) on improving HFD-induced emotional changes, and tried to identify their different mechanisms. The intraperitoneal glucose tolerance test (IPGTT) was used to evaluate glucose control ability; and behavior tests including open field tests (OFT), forced swimming tests (FST), tail suspension tests (TST) and sucrose preference (SPT), were then performed to evaluate emotional changes. Serum samples were collected for the LC-MS based metabolomics analysis to explore the emotional-related differential compounds; we then evaluated the effect of the drugs. The abnormal serum metabolic profiling and emotional changes caused by HFD in mice was alleviated by SMV treatment, whereas BZ only affected the emotional disorder. The improvement of cannabinoid analogues and then produced influences on the endocannabinoid system, which may be a potential mechanism SMV action. BZ promoted tryptophan-serotonin pathway and inhibited tryptophan-kynurenine pathway, which may be its mechanism of action. Here, we proposed a shed light on the biological mechanisms underlying the observed effects, and identified an important drug candidate for the treatment of emotional disorders induced by HFD.

## Introduction

According to a systematic and global analysis in children and adults from 1980–2013, the overweight and obese population has reached nearly 2.1 billion wordwide^1^. Accumulated clinical studies have shown that depression and diabetics are frequently comorbid^2^. Indeed the development of type 2 diabetes mellitus (T2DM) might be an underlying cause of depression, as the prevalence of depression in T2DM patient is two times the number of people without T2DM^3^. It was recently reported that HFD affected animal emotional status and cognitive functions, such as depression^4, 5^ and Alzheimer’s disease (AD)^6, 7^. Hence, it is a challenge for scientist to explore therapeutic treatments for HFD-induced emotional disorders.

SMV and BZ are known as the lipid-lowing agents, which are widely used in clinical therapeutics, though their mechanisms of action are different. As HMG-CoA (3-hydroxy-3-methylglutaryl-coenzyme A) reductase inhibitors, statins can effectively decrease blood cholesterol levels^8^. Recently, SMV has been reported to show some cholesterol-independent effects that include anti-inflammatory, anti-oxidative and neuro-protective properties^9^. In contrast, it can cross the blood-brain barrier (BBB) and exert some serious adverse effects on neurons as a lipophilic drug^10^. Clinical studies regarding the effects of statins on depression are incongruous, and statistical data show that the use of SMV after a cardiac intervention is associated with a reduced risk of subsequent depression^11^.

BZ, a peroxisome proliferator activated receptor α (PPARα) agonist, showed a strong anti-inflammatory effect^12^. Thiazolidinedione is reported to have some beneficial effects on some neurological diseases, such as AD, Amyotrophic lateral sclerosis (ALS) and Parkinson’s disease (PD)^13–21^. Moreover, it is interesting that PPAR agonists exert neuro-protective effects by reducing oxidative stress and inflammation^22^. Dumont *et al*. showed that BZ treatment could improve behavioral deficits and tau pathology in P301S mice; thus, BZ is considered a promising agent for neurodegenerative diseases associated with tau pathology^23^. To date, there are no reports on the effect of BZ on anxiety- and depression-like behaviors.

The existing research mostly focuses on the effect of drug treatment on behavioral, pathological and biochemical changes^24–27^. However, little is known about metabolite changes in HFD-induced-emotional disorder mice with drug intervention. In the current study, an LC MS-based serum metabolomics analysis coupled with behavioral tests was performed to elucidate the underlying mechanism of drug intervention (BZ and SMV) for improving emotional changes induced by HFD.

## Results

### Effects on metabolic parameters

**Figure 1:**
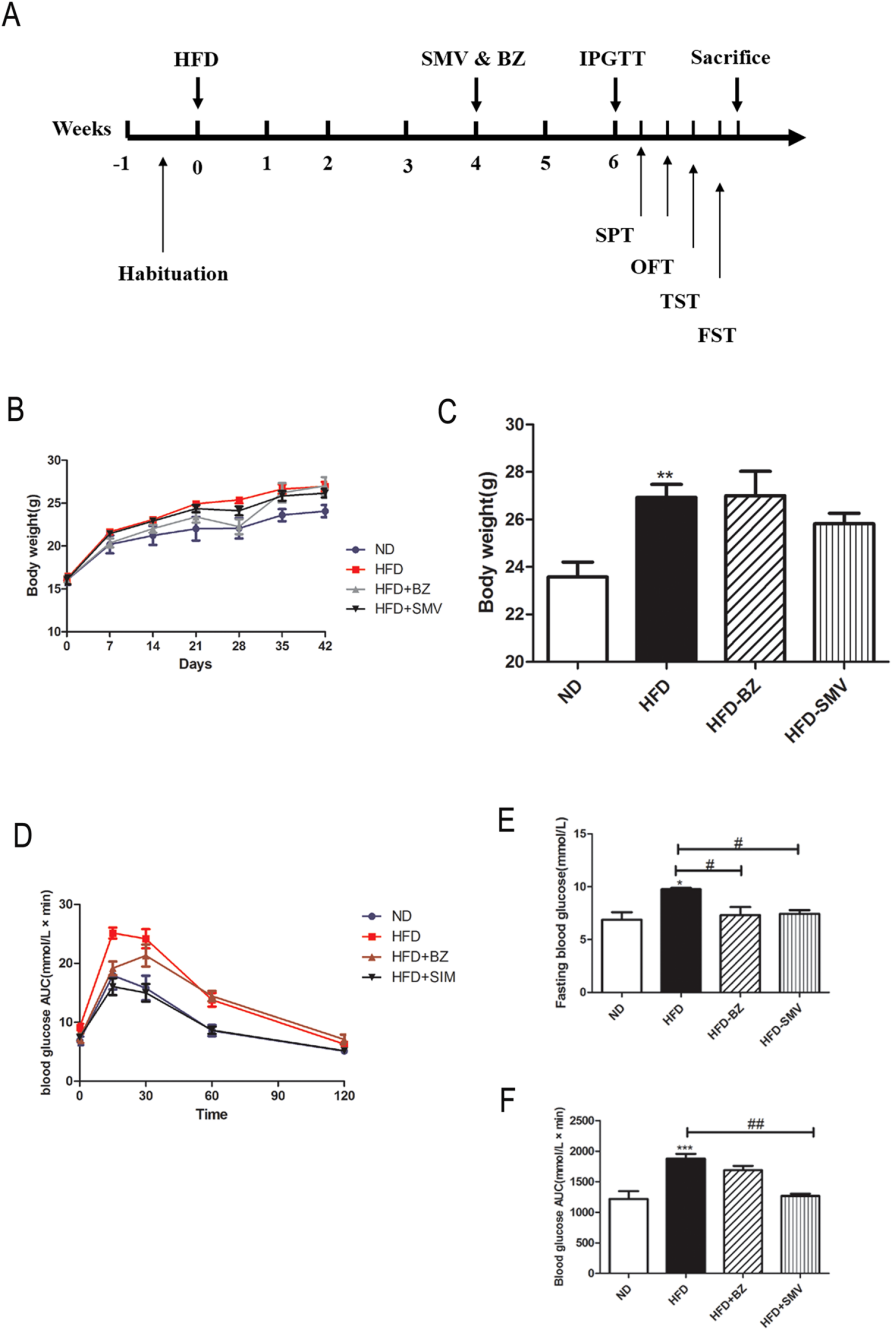
The effects of high fat diet and drug intervention (BZ&SMV) on metabolic parameters. Experimental procedure. Mice were fed with ND or HFD for 4 weeks and then received either SMV (5 mg/kg/day, i.p.), BZ (75 mg/kg/day, i.p.) or vehicle treatment (CMC-Na, 0.5%, i.p.) (**A**), Body weight change (**B**), final body weight (**C**), blood glucose levels during an IPGTT (**D**), fasting glucose levels (**E**) and AUC of the glycaemia over 120 min (**F**). *P < 0.05 ; **P < 0.01; ***P < 0.001 vs. ND group. ^#^ *P* < 0.05; ^##^ *P* < 0.01 vs. HFD group.

We first compared the animal body weights across the experimental time points as shown in Fig. 1B,C. We found that the animals fed with a HFD showed significant high body weights compared to the normal diet group (*P* < 0.01). However, BZ (75 mg/kg/day) and SMV (5 mg/kg/day) treatment did not result in any significant effects on body weight (*P* > 0.05).

Fasting blood-glucose and IPGTT were performed at the end of 6th week, and the blood glucose levels during the IPGTT are shown in Fig. 1D. The HFD group showed significant high fast blood glucose (FBG) levels compared to the ND group (*P* < 0.05), and both two drugs can significantly reduce FBG levels (*P* < 0.05) as is shown in Fig. 1E. And the areas under the curve (AUC) for the glycaemia over the 120 min time period is represented in Fig. 1F; the AUC in the HFD group significantly increased compared to the ND group (*P* < 0.001). SMV (5 mg/kg/day) treatment can significantly improve the glucose tolerance, though there were no significant differences in the BZ (75 mg/kg/day) treatment group. Overall, an HFD diet increased body weights, fasting hyperglycemia and glucose tolerance over the course of the experiment. SMV improved HFD-induced fasting hyperglycemia and glucose tolerance in mice, while BZ was effectively only for fasting hyperglycemia.

### Effects on emotionality parameters

The OFT is widely used to determine an animal’s locomotor activities and exploratory behaviors. TST and FST can effectively reflect behavioral despair through the assessment of immobility time. Additionally, mouse anhedonia is presented by SPT.

**Figure 2:**
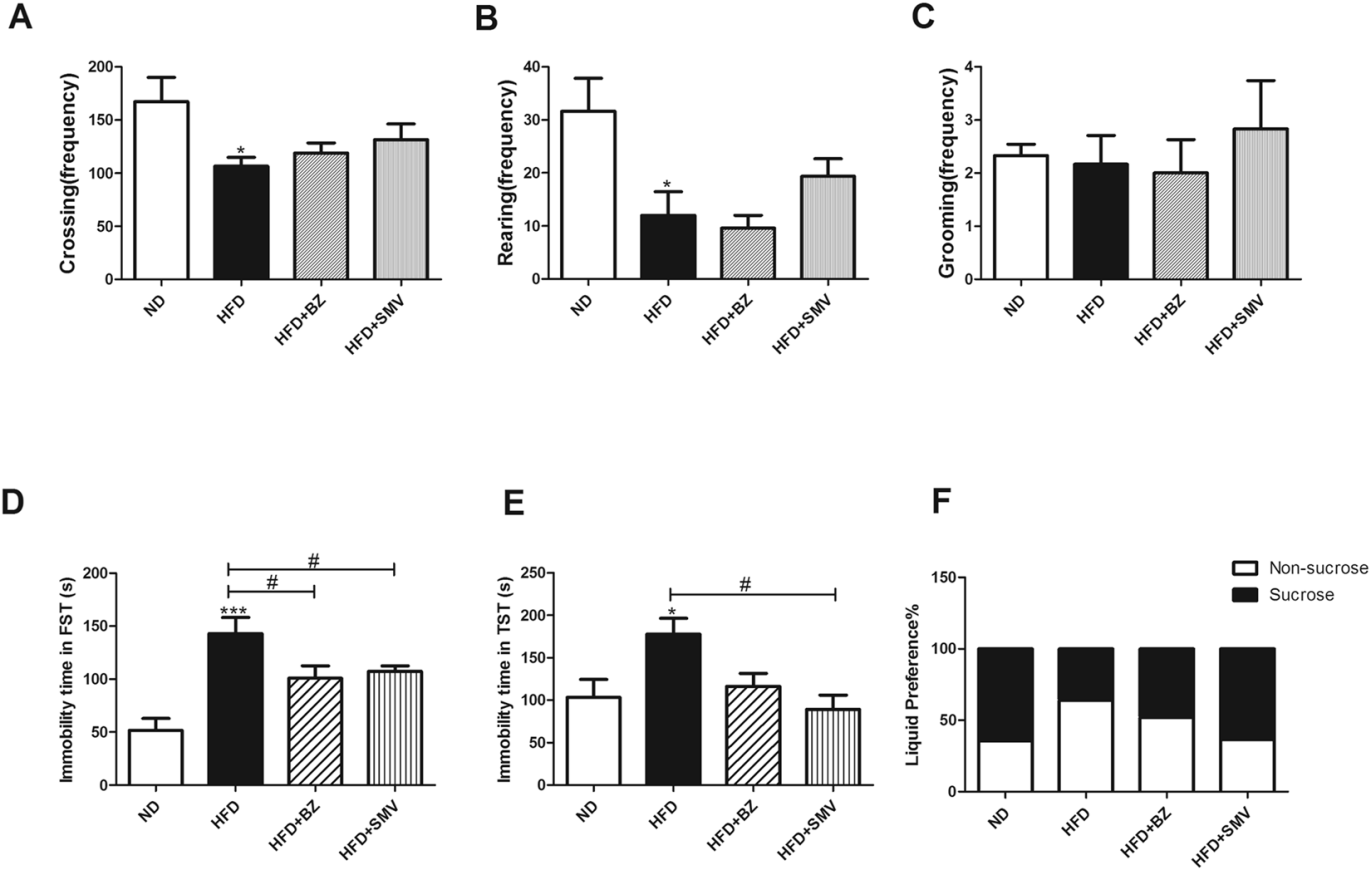
The effects of high fat diet and drug intervention (BZ&SMV) on the emotionality parameter mice. Number of crossings (**A**), rearings (**B**) and groomings (**C**) in the OFT. Immobility time (s) in the FST (**D**) and TST (**E**). The liquid preference (percentage) in the SPT (**F**). *P < 0.05; **P < 0.01; ***P < 0.001 vs. ND group. ^#^ *P* < 0.05 vs. HFD group.

The OFT result in Fig. 2A–C showed that, the number of crossings and rearings were significant reduced in the HFD group compared to the ND group (*P* < 0.05). However, there were no significant changes in the number of groomings. SMV (5 mg/kg/day) and BZ (75 mg/kg/day) showed no difference in locomotor activity.

Figure 2D,E show that, there was a significant increase in the immobility time in HFD group in both FST (*P* < 0.001) and TST (*P* < 0.05) experiment compared to the ND group. SMV (5 mg/kg/day) could significantly reduce the immobility time in both the FST and TST experiments (*P* < 0.05). However, the BZ (75 mg/kg/day) could only reduce the immobility time significantly in the FST experiments (*P* < 0.05), and showed no significant differences in the TST experiment (*P* > 0.05). The above data suggests the antidepressant potential of SMV and BZ in HFD-exposed animals by reducing immobility time, and that SMV has a better effect.

The liquid preference in the SPT experiment in Fig. 2F showed a reduction in sucrose consumption in the HFD group compared to the ND group. SMV treatment (5 mg/kg/day) could improve the mean mouse anhedonia condition, whereas BZ treatment (75 mg/kg/day) showed no effects.

### Metabolic profiling of serum from the HFD and ND groups

**Figure 3:**
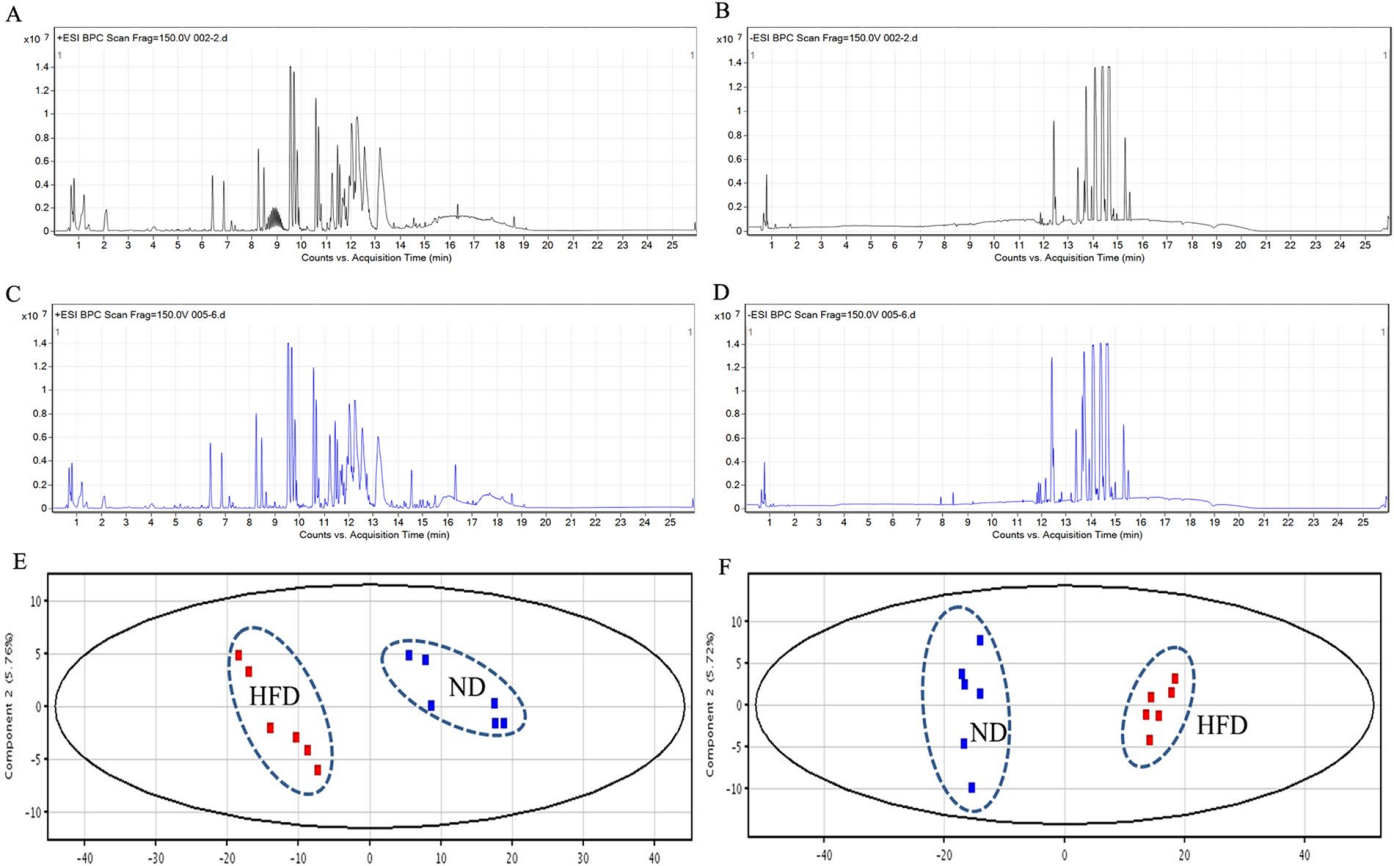
Metabolic profiling of serum samples in HFD and ND mice. The typical base peak ions chromatograms (BPCs) of serum samples from HFD (**A**,**B**) and ND (**C**,**D**) in ESI positive (**A**,**C**) and negative mode (**B**,**D**). Score plots of PCA analysis for serum samples from [HFD (▪), ND (▪)] groups in ESI positive mode (**E**) and negative mode (**F**).

The typical total ion chromatograms (TICs) for HFD and ND group serum samples in both ESI positive and negative modes are shown in Fig. 3A–D. It is clear that there was an observed difference in the number and intensity of spectrum peaks between the HFD and ND groups. These differences could found between the groups, but could also exist between different individuals in the same group. To obtain more subtle differences among these complex data sets, a pattern recognition approach, such as principal component analysis (PCA) was performed. PCA score plots (Fig. 3E,F) showed a clear separation between the HFD and ND groups in both ESI positive and negative modes, indicating that HFD could lead to significant variations in the serum metabolic profiling.

The Mass Profiler Professional (MPP) software was used to explore the differential compounds between the HFD and ND groups (*P* < 0.05 and Fold change >1.5). The differential compounds were identified using “ID browser” to search in METLIN Database, and then searched the matches of the features and related metabolic pathway in KEGG and HMDB database. The differential compounds and related metabolic pathway are summarized in Table 1. These compounds could be roughly divided into the following categories: (1) Fatty acid synthesis and metabolism, (2) Sphingosine metabolism, (3) tryptophan metabolism, (4) biotin metabolism, and (5) pyrimidine metabolism. Among them, the levels of LysoPE (20:3 (11Z, 14Z, 17Z)/0:0), Trihexosylceramide (d18:1/12:0) and DL-Phenylalanine were significantly increased by HFD. In contrast, the levels of LysoPE (0:0/16:1(9Z)), Eicosapentaenoic Acid ethyl ester, Palmitoyl Ethanolamide (PEA), PE (20:0/18:3 (9Z, 12Z, 15Z)), Oleoyl Ethanolamide (OEA), Linoleoyl Ethanolamide (LEA), N,N-Dimethylsphingosine, 2′-Deoxyuridine, Bisnorbiotin, LysoPE (22:5 (4Z, 7Z, 10Z, 13Z, 16Z)/0:0), PE (20:3(5Z, 8Z, 11Z)/20:2 (11Z, 14Z)) and Serotonin were decreased.

**Table 1:** The differential metabolites in serum samples from [HFD, ND] groups in ESI Positive and Negative Mode.

Compound^a^	P(corr)	Regulation	Metabolic pathway
**ESI Positive mode**			
LysoPE(20:3 (11Z, 14Z, 17Z)/0:0)	0.00279	↑	Fatty acid biosynthesis metabolism
Trihexosylceramide (d18:1/12:0)	0.00264	↑	Fatty acid biosynthesis metabolism
LysoPE(0:0/16:1(9Z))	0.00264	↓	Fatty acid biosynthesis metabolism
**ESI negative mode**			
Eicosapentaenoic Acid ethyl ester	0.014419	↓	Fatty acid biosynthesis metabolism
Palmitoyl Ethanolamide	7.92E-04	↓	Fatty acid biosynthesis metabolism
PE(20:0/18:3 (9Z, 12Z, 15Z))	0.002736	↓	Fatty acid biosynthesis metabolism
Oleoyl Ethanolamide	1.22E-04	↓	Fatty acid biosynthesis metabolism
Linoleoyl Ethanolamide	1.67E-04	↓	Fatty acid biosynthesis metabolism
N,N-Dimethylsphingosine	0.01712	↓	Sphingosine Metabolism
2′-Deoxyuridine	0.012431	↓	Pyrimidine metabolism
Bisnorbiotin	0.001095	↓	Biotin metabolite
DL-Phenylalanine	0.014274	↑	Phenylalanine, tyrosine and tryptophan biosynthesis
LysoPE(22:5 (4Z, 7Z, 10Z, 13Z, 16Z)/0:0)	0.002114	↓	Fatty acid biosynthesis metabolism
PE(20:3 (5Z, 8Z, 11Z)/20:2 (11Z, 14Z))	0.014677	↓	Fatty acid biosynthesis metabolism
Serotonin	1.85E-02	↓	Tryptophan metabolism

Statistical significance levels were determined by ANOVA test. Only metabolites with p-values of less than 0.05 were deemed to be statistically significant.

**Figure 4:**
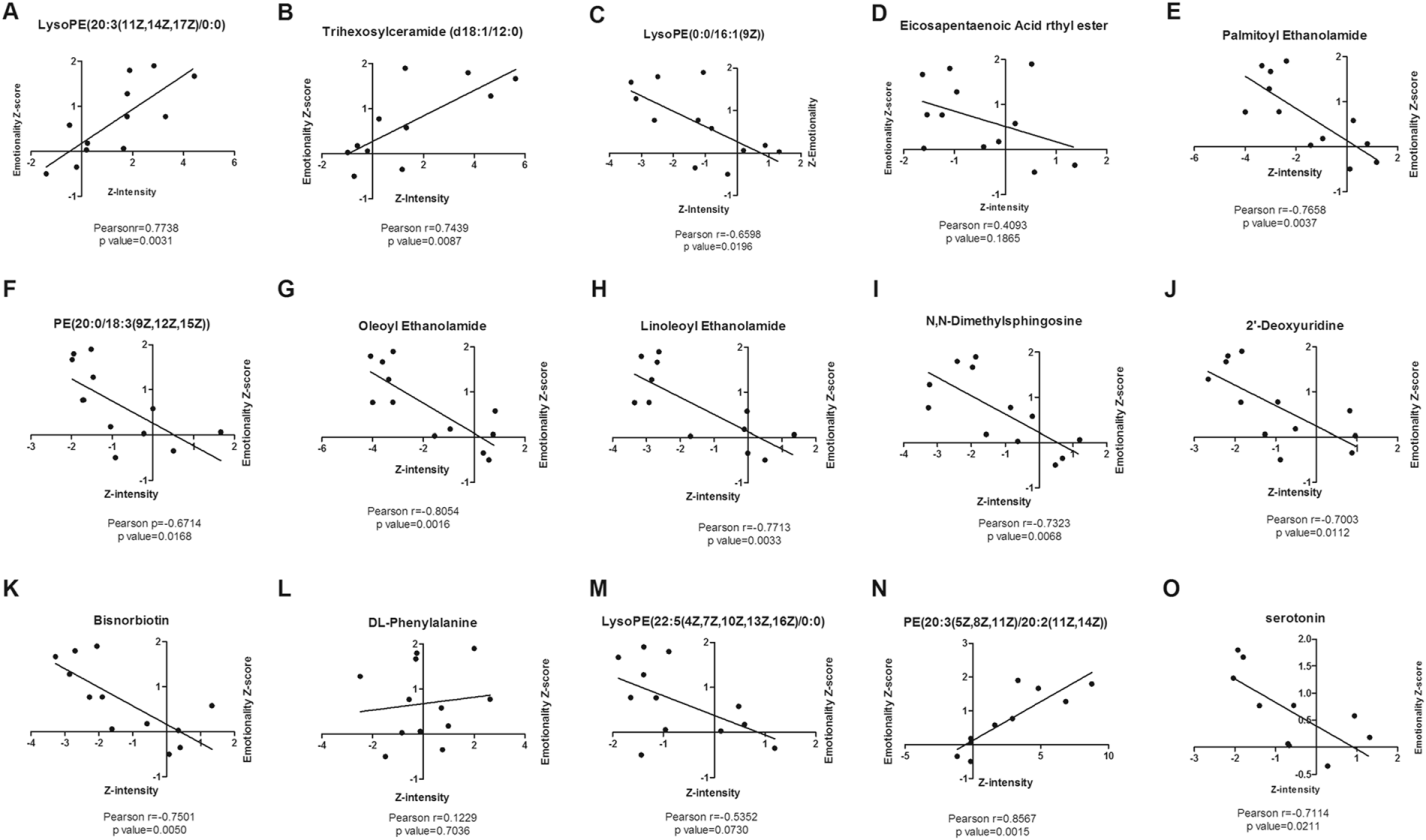
The correlation between the differential compound intensity Z-score and Emotionality Z-score. Emotionality Z-score included: the number of grooming, rearing and crossing in the OFT, the immobility time in the TST and FST.

We next aimed to explore the relationships between the differential compounds and the changes in the emotional parameters. As shown in Fig. 4, except for Eicosapentaenoic Acid ethyl ester (Pearson r = 0.4093, *p* value = 0.1865), DL-Phenylalanine (Pearson r = 0.1129, *p* value = 0.7036) and LysoPE (22:5 (4Z, 7Z, 10Z, 13Z, 16Z)/0:0) (Pearson r = −0.5352, *p* value = 0.0730)), the remaining 12 compounds showed a significant correlation with the changes in the emotional parameters. Among these emotional-related compounds, a significant positive correlation between the LysoPE (20:3 (11Z, 14Z, 17Z)/0:0) (Pearson r = 0.7738, *p* value = 0.0031), Trihexosylceramide (d18:1/12: 0) (Pearson r = 0.7439, *p* value = 0.0087), PE(20:3 (5Z, 8Z, 11Z)/20:2 (11Z, 4Z)) (Pearson r = 0.8657, *p* value = 0.0015) intensity and emotionality Z-scores. Whereas a significant negative correlation was observed between the Z-scores of LysoPE(0:0/16:1(9Z)) (Pearson r = −0.6598, *p* value = 0.0196), PEA (Pearson r = −0.7658, *p* value = 0.0037), PE(20:0/18:3 (9Z, 12Z, 15Z)) (Pearson r = −0.6714, *p* value = 0.0168), OEA (Pearson r = −0.8054, *p* value = 0.0016), LEA (Pearson r = −0.7713, *p* value = 0.0033), N,N-Dimethylsphingosine (Pearson r = −0.7323, *p* value = 0.0068), 2′-Deoxyuridine (Pearson r = −0.7003, *p* value = 0.0112), Bisnorbiotin (Pearson r = −0.7501, *p* value = 0.0050), Serotonin (Pearson r = −0.7114, *p* value = 0.0211) intensity and emotionality Z-scores.

### Effects of BZ & SMV on serum metabolic profiling

**Figure 5:**
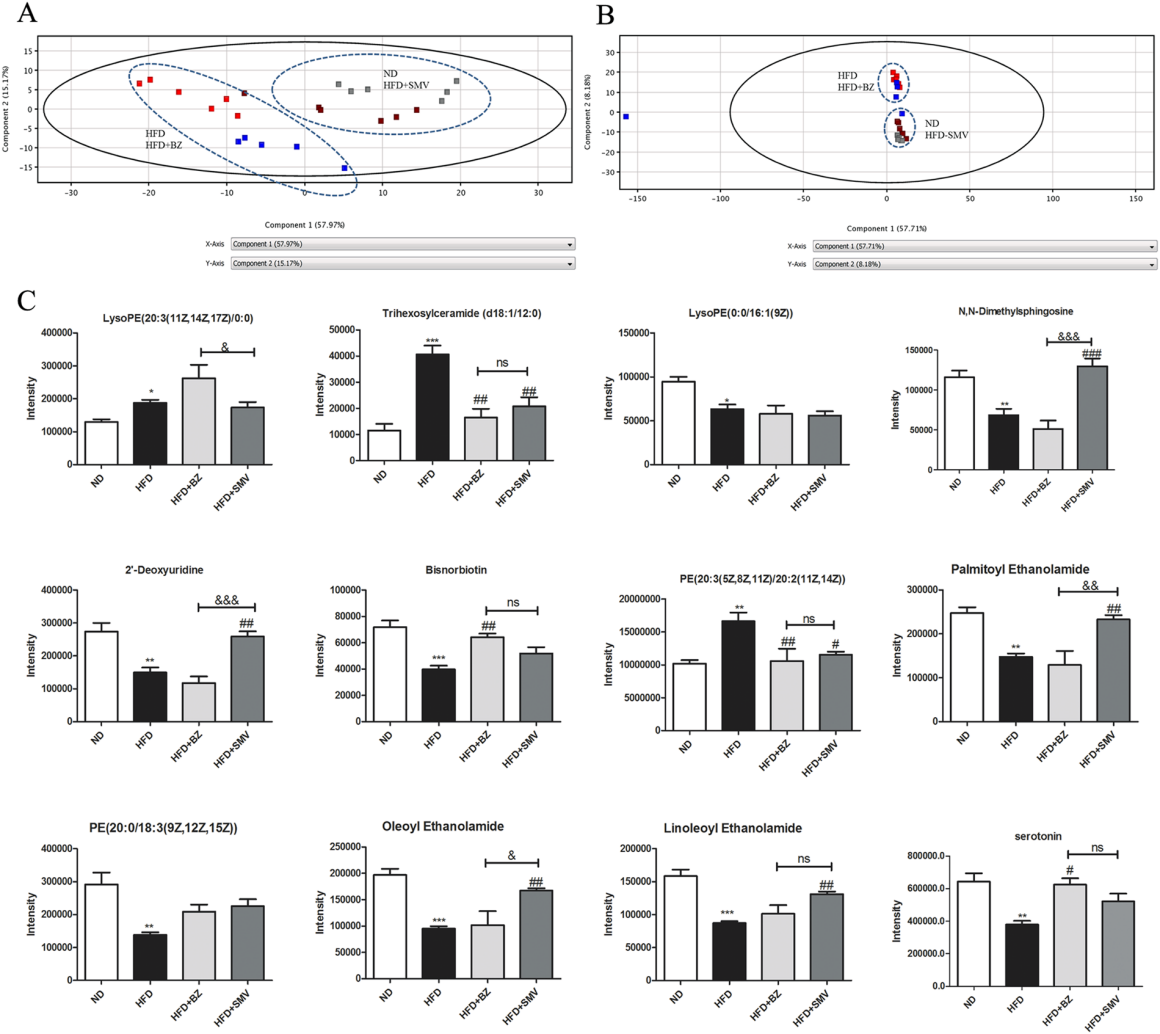
The effect of high fat diet and drug intervention (BZ & SMV) on the serum profiling in mice. The score plot of PCA analysis for serum samples from [HFD (▪), ND (▪), HFD + BZ (▪), HFD + SMV (▪)] groups in ESI positive mode (**A**) and negative mode (**B**). Emotional-related differential compounds (**C**). *P < 0.05; **P < 0.01; ***P < 0.001 vs. control group. ^#^ *P* < 0.05; ^##^ *P* < 0.05 ; ^###^ *P*<0.001 vs. HFD group. ^&^ *P* < 0.05, ^&&^ *P* < 0.01 between BZ group and SMV group.

The serum metabolic profiling presented in Fig. 5A,B, the position of SMV treatment group was near to the ND group, suggesting that SMV can reverse the changed metabolic patterns caused by HFD. However, BZ treatment group was near to HFD group, indicating that BZ did not affected the changed metabolic patterns caused by HFD. Combined with the emotionality parameters, the above results indicated that SMV could ameliorate the abnormal metabolic and emotional statuses, while BZ could only improve the depression-like symptom, and had no effect on the metabolic pattern.

To explore the different mechanisms between BZ and SMV for the treatment of HFD-induced emotional changes, the intensities of the emotional-related compounds were compared as shown in Fig. 5C. The results indicated that SMV treatment showed a tendency of bring the level of 7 emotional related compounds (Trihexosylceramide (d18:1/12:0), PEA, OEA, LEA, N, N-Dimethyl-sphingosine, 2′-Deoxyuridine, PE (20:3 (5Z, 8Z, 11Z)/20:2 (11Z, 14Z))) to normal. However, BZ could only bring the levels of 4 compounds (Trihexosylceramide (d18:1/12:0), Bisnorbiotin, PE (20:3 (5Z, 8Z, 11Z)/20:2 (11Z, 14Z)), Serotonin) to normal.

### Effects on tryptophan (TRP)-related compound levels

BZ treatment could tend to bring the levels of serotonin to normal. TRP is known as a serotonin (5-HT) precursor, and is metabolized in the kynurenine (KYN) pathway. To explore the different mechanisms of two drugs treatments, the metabolites in the TRP metabolic pathway were examined.

**Figure 6:**
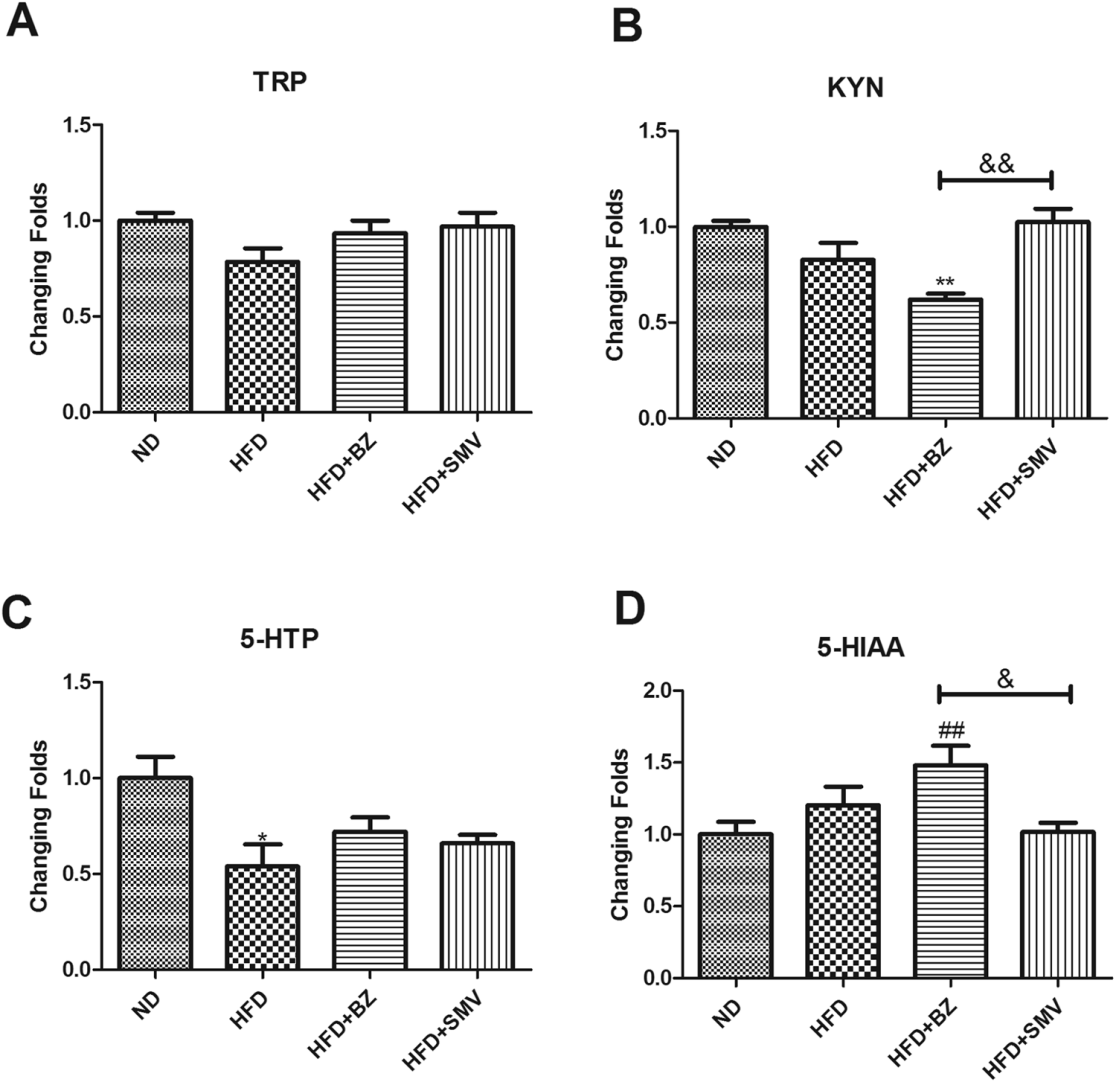
The effect of high fat diet and drug intervention (BZ & SMV) on serum tryptophan-related compound contents. *P < 0.05 vs. ND group. ^#^ *P* < 0.05; ^##^ *P* < 0.05 vs. HFD group. ^&^ *P* < 0.05 between BZ group and SMV group.

As shown in Fig. 6, the HFD and drug treatments showed no differences in serum TRP levels (*P* > 0.05). BZ (75 mg/kg/day) treatment significantly decrease the serum KYN (*P* < 0.01) levels and increased the 5-HIAA levels (*P* < 0.01) in the HFD group. This result might indicate that BZ can promote the conversion of TRP into 5-HT and inhibit the metabolism of TRP to KYN. However, there were no significant differences in the SMV (5 mg/kg/day) treatment group.

## Disscussion

Obese animals with endocrine abnormalities usually show symptoms of hyperglycemia and insulin resistance^28^. Surwit *et al*. first reported that HFD can induce a type 2 diabetic model in the early 1980’s, and the model is more efficient in C57BL/6 J mice compared with other strains^29^. Later, in 2004, Winzell *et al*. validated this model in a large number of C57BL/6J mice, the mice showed normal fasting blood glucose and insulin levels but impaired glucose tolerance after short-term high fat diet feeding, suggesting that HFD is an efficient model for IGT and early type 2 diabetics in C57BL/6J mice^30^. After short-term HFD, the impaired glucose decreased in mice, even if they showed normal glucose and insulin levels^31^. In agreement with the previous study^32^, Fig. 1 shows that high fat diet induced a decreasing acute blood glucose control ability and a typical glucose intolerance. SMV administration can significantly ameliorate the glucose intolerance, while BZ showed no significant differences.

HFD can induce anxiety- and depression-like behaviors in mice^33^. Zemdegs *et al*. integrated the metabolic and emotional parameters using Z-score, and found that there was a significant positive correlation between metabolic and emotional changes after both 12 and 16 weeks of HFD^34^. Can Ö D *et al*.^35^ found that 4-weeks of 5 mg/kg of SMV can significantly change the depression-like behaviors, though there were no difference in animal locomotor activity. So far, effect of BZ in improving HFD- induced emotional disorders has not been reported. As is shown in Fig. 2, HFD mice displayed typical depression symptoms, including reducted spontaneous activities and despair behaviors. After BZ and SMV intervention, the despair behaviors induced by HFD improved, but not spontaneous activity, which suggested antidepressant effects by the two drugs.

As an important indicator of peripheral circulation that can reflect the real-time changes in various life activities accurately, serum has become a hot research topic in metabolomics studies^36^. An LC MS-based serum metabolomics analysis was performed to investigate the metabolic changes in mice due to the HFD and drug intervention. HFD led to significant variations in serum metabolic profiling in mice, which indicated metabolic disorders in the HFD group mice (Fig. 3), as 15 compounds were found to significantly change between the HFD and ND groups (Table 1). Moreover, taking high fat diet induced emotional change as the research topic, it is essential to explore the relationship between these differential compounds and the emotional disorder in the corresponding individuals. In total, 12 compounds showed a significant correlation with the emotional changes (Fig. 4). It is considered feasible to validate the antianxiety or antidepressant effects of drug treatment by examining the expression levels of emotional-related differential compounds.

Among the 12 emotional-related compounds, Lysophosphatidylcholines are the main components of oxidized density lipoprotein (ox-LDL). Almost 40% of phosphatidylcholines could be transformed into lysophosphatidylcholines during the oxidation of LDL-C, which is known to affects inflammation response and oxidative stress^37^. The increased serum LysoPE levels suggest an increasing oxidative stress in the HFD group. As a type of glycosylsphingolipid, Trihexosylceramide (d18:1/12:0) accumulates in tissues due to defects in ceramide trihexosidase and considered to be the cause of angiokeratoma corporisdiffusum^38^. Additionally, PEA, OEA and LEA have been identified as structural analogs of endocannabinoids (ECs). The endocannabinoid system is known to play an important role in some neurological diseases such as schizophrenia, stroke, and AD^39–41^. Although these EC-like ligands cannot bind to cannabinoid receptors with high affinity, they may influence endocannabinoid function via competition for catabolic enzymes^42^. It has been reported that EC-like ligands show anti-inflammatory, analgesic, anticonvulsant and neuroprotective properties^43–46^. Moreover, OEA can regulate feeding and body weight^47^. The EC-like ligands were significantly reduced in the HFD group, which may indicate the disorder of fatty acid amide biosynthesis and metabolism, thereby affecting the endocannabinoid systems that led to the change in inflammation, neuroprotection and feeding behaviors.

Interestingly, the results showed that 5-HT significantly decreased in the HFD group compared with the ND group, and that there is a strong negative correlation between serum 5-HT levels and emotional changes. In fact, 5-HT is an important monoamine neurotransmitter that is closely linked to moods disorders and is synthesized by TRP in the brain and intestine^48^. Indeed, the balance in monoamine neurotransmitters in the central and peripheral nervous systems is closely related with mood disorders^49^. The above result are consistent with previous research on depression animal models^50, 51^; however, the changes in 5-HT in the HFD-induced obesity animal model is complicated. Kim H J *et al*. reported that 5-HT levels significantly increased^52^. In contrast, Kim M *et al*. study found that 5-HT levels decreased significantly, and was negatively correlated with body weight in the corresponding individuals^53^.

Previous studies on clinical trials and animals support the antidepressant effects of SMV, and in our studies, it is demonstrated that BZ and SMV do improve the emotional changes in mice induced by HFD. Nevertheless, the potential drug mechanisms of action are still not clear. Serum metabolic profiling results showed that SMV can reverse the abnormal metabolic patterns caused by HFD, but not BZ (Fig. 5A,B). To further examine the different BZ and SMV mechanisms of action, the intensities of emotional-related compounds were examined and then compared (Fig. 5C). These data suggest that SMV may improve fatty acid amide biosysthesis and metabolism; as well as fatty acid metabolism and necleoside metabolism. As the EC-like ligands, OEA, LEA and PEA exerts effects by influence the endocannabinoid function via competition catabolic enzymes. BZ can significantly improve 5-HT levels and some metabolites involved in fatty acid biosysthesis metabolism.

Considering the important role of 5-HT in emotional disorders, and the above results suggested that only the BZ can significantly improve serum 5-HT levels, we further explored the upstream and down stream metabolites of 5-HT, and tried to identify the different mechanisms of action for the two drugs (Fig. 6). TRP is the precursor of 5-HT *in vivo* and is associated with various physiological functions in different tissues. KYN produced by TRP in immune cells plays a key role in regulating immune responses in infection and inflammation^54^. Almost 95% of TRP *in vivo* converted to KYN by the action of the largely hepatic-based enzyme, tryptophan-2, 3-dioxygenase (TDO) or the ubiquitous indoleamine-2, 3-dioxygenase (IDO)^55^. TDO is mainly located in the liver and is induced by glucocorticoid or tryptophan levels, whereas IDO is widespread in numerous tissues and activated by certain inflammatory stimuli, among which IFN-γ is the main inducer^56^. Due to the certain intake of tryptophan from dietary proteins, the TRP-KYN metabolism is usually mediated by IDO *in vivo* ^57^. Most of the KYN in the CNS is derived from peripheral blood; KYN can be transported across the BBB and participate in the synthesis of nervous excitation metabolites in the CNS. Thus, increasing KYN plasma can reflect CNS status^58^. The excessive activation of the TRP-KYN metabolic pathway may cause a reduction of 5-HT synthesis and an increase in nervous excitation metabolites in the KYN pathway, which eventually influence the central and peripheral nervous systems. BZ treatment can promote the TRP- 5-HT pathway and inhibit the TRP-KYN pathway. However, there were no significant differences in TRP metabolism after SMV treatment. As a nonselective agonist of the peroxisome proliferator activated receptor, BZ has a strong anti-inflammatory effect^12^. Accordingly, abnormal activation of the TRP-KYN pathway may reflect the inflammation levels *in vivo* ^56^. The anti-inflammatory effect is a potential mechanism for the treatment of HFD-induced emotional changes.

## Conclusions

**Figure 7:**
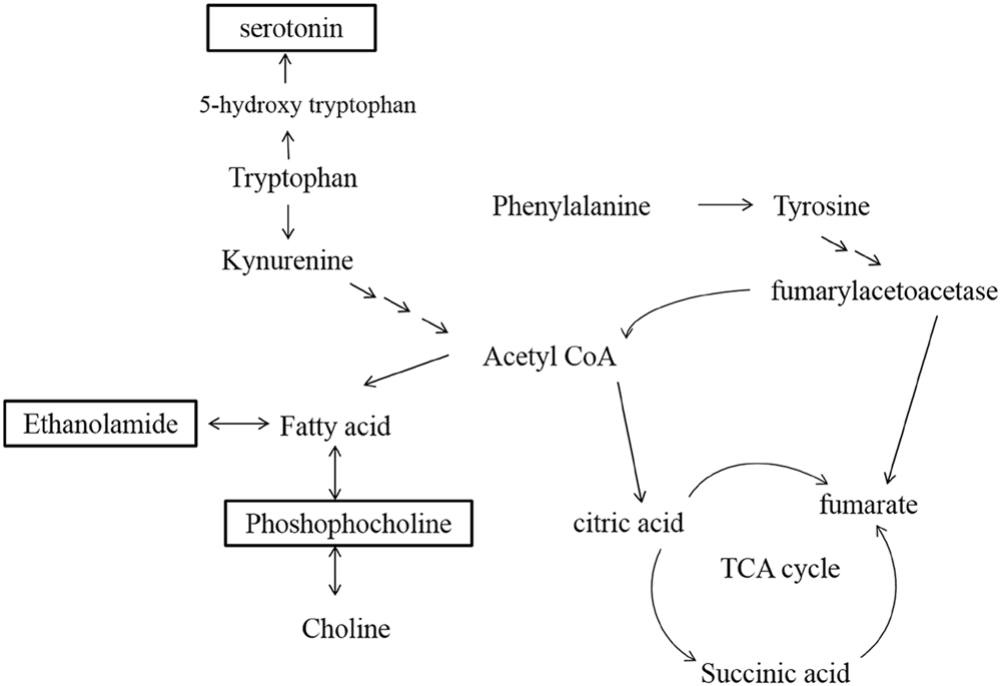
The perturbed metabolic pathways and relevant pathways leading to high fat diet -induced emotional changes.

In summary, we combined behavioral assessments and serum metabolomics methods to study the different mechanisms of action for BZ and SMV in HFD-induced emotional changes. The perturbed and relevant pathways induced by HFD are showed in Fig. 7, SMV and BZ can both prevent the development of emotional disorders caused by HFD, but SMV is more effective. The improvement of cannabinoid analogues thereby affecting the endocannabinoid system occurring during SMV treatment. Otherwise BZ promotes the TRP-5-HT pathway and inhibits the TRP-KYN pathway. The above results may provide new ideas and scientific evidence for the further development of new drug indications and clinical medications. SMV and BZ might be effective for diet-induced metabolic disorder patients suffering from emotional disorders. The effect of these two drugs on metabolism in the central nervous system and their exact mechanisms of action remain to be explored further.

## Material and Methods

### Chemicals and Reagents

The normal and high fat diets were purchased from Jiangsu province Cooperative Medical Biological Engineering Co., Ltd (Jiangsu province, China). D-Glucose was purchased from Sigma-Aldrich (St. Louis, MO, USA). Bezafibrate was purchased from Jiangsu WanGao Pharmaceutical Co., Ltd. (Jiangsu province, China). Simvastatin was purchased from Harbin Pharmaceutical Group Sanjing Mingshui Pharmaceutical Co. Ltd. (Heilongjiang Province, China). Acetonitrile (LC-MS grade) was purchased from Merck (Darmstadt, GER), Formic acid and ammonium fluoride were purchased from Fisher (Marshalltown, USA). The distilled water was prepared using a Millipore Milli-Q purification system (Millipore, Billerica, MA).

### Animals and Treatment

The experiments performed on animals were in accordance with protocols approved by the Animal Experimentation Ethics Committee of Chinese Pharmaceutical University (Approval ID: SCXK (Su) 2011–0003).

Twenty-four male C57BL/6J mice (5–6 weeks old, weight 15–17 g) were obtained from Changzhou Cavensla Laboratory Animal Co., Ltd. (Jiangsu province, China). The care and handling of the mice were in accordance with the Specific Pathogen Free standards. Animals were housed under a reversed 12-h light/12-h dark cycle at constant temperature (22 ± 1 °C), and animals had free access to food and water during the experiment. After 1- week of habituation, the mice were randomly divided into the following four groups: (1) normal diet group (ND, n = 6), (2) high fat diet group (HFD, n = 6), (3) high fat diet + bezafibrate group (HFD+BZ, n = 6), (4) high fat diet + simvastatin group (HFD+SMV, n = 6). The animals were fed with an ND or HFD during the first 4 weeks, and then received drug intervention during the last 2 weeks. SMV (5 mg/kg/day) and BZ (75 mg/kg/day) treatments were administered by oral gavage once per day. The ND groups received equal volumes of 0.5% CMC-Na solution. The detailed experimental procedure is shown in Fig. 1A.

### Fasting blood-glucose and intraperitoneal glucose tolerance test (IPGTT)

All of the animals were fasted overnight, and fasting blood-glucose was taken before treatment with D-glucose (2 g/kg) intraperitoneal injection. The blood samples were taken at 15, 30, 60, and 120 min after glucose administration. Blood glucose was measured using a glucose meter (Bayer medicine health care Co., Ltd.). To reflect the glucose tolerance, the areas under the glucose concentration time curve of the glucose concentration after the glucose injection in 2 hours was calculated.

### Behavioral tests

#### Open field test (OFT)

Each mouse was placed into the center of the test box (100 × 100 × 40 cm, with its floor divided into 25 squares) for 1 min for adaptation and then allowed to explore the area for 5 min. The number of crossings, rearings and groomings were recorded and measured. After each test, the box was cleaned with 70% alcohol before the next animal test.

#### Tail suspension test (TST)

The mice were placed with their heads hanging down with tape wrapped around the tail on a bracket 50 cm high from the ground. The immobility time was recorded with a stopwatch over the 5 min test.

#### Forced swim test (FST)

The mice were placed separately in a glass beaker (50 cm in height, 20 cm in diameter and 30 cm in water depth), with water temperature controlled at 24 ± 2 °C. The experiment procedure included the following two stages: (1) 15 min of swimming pretest and (2) 5 min swimming formal test after 24 h. The immobility time were recorded with a stopwatch over the 5 min formal test.

#### Sucrose preference test (SPT)

Animals were first trained to drink 1% sucrose solution from two bottles (48 h before the formal experiment). Twenty-four hours later, the animals were allowed free access to 1% sucrose and water solution in two bottles. To avoid bottle side preference, the two bottles were switched. The amounts in the two bottles were measured after 24 h and the sucrose preference was calculated according to the following formula:

$${\rm{Sucrose}}\,{\rm{preferencec}}( \% )={\rm{sucrose}}\,\mathrm{consumption}/({\rm{sucrose}}+{\rm{water}}\,{\rm{consumption}})\times 100 \% $$

### Serum sample pretreatment

Serum samples (100 μL) were mixed with 400 μL cold acetonitrile and then vortexed for 3 min to precipitate the protein. After centrifugation (13300 rpm at 4 °C for 15 min), the supernatant was transferred to another vial and then dried at room temperature using a Thermo SPD1010 SpeedVac Kit (Thermo, USA). The dried residues were dissolved in 50% acetonitrile and ultrasonicated for 15 min to dissolve the samples. After centrifugation (13300 rpm at 4 °C for 15 min), the supernatant was transferred into the autosampler vials and a 2 μL aliquot was injected for LC-MS analysis.

### LC-MS Analysis

Serum metabolic profiling was performed using a 1260 Rapid Resolution Liquid Chromatography (RRLC) system coupled to a 6530 quadrupole time of flight (Q-TOF) MS (Agilent, Santa Clara, CA).The separations was achieved using a Poroshell SB-AQ column (2.1 × 100 mm × 2.7 μm, Agilent) with the column temperature held constant at 40 °C and a pump flow rate of 0.4 mL/min. In the positive mode analysis, the mobile phase system was composed of A (water with 0.1% formic acid) and B (acetonitrile). In the negative mode analysis the mobile phase system included A (1 mM ammonium fluoride in water) and B (acetonitrile).The gradient elution program was as follows: 0–2 min, 0% B; 2–20 min, 0–100% B; 20–25 min, 100% B.

The electrospray ionization (ESI) source was set in positive and negative mode with the data being collected between m/z 50–1000. For the positive ionization mode, the MS conditions were as follows: The flow rate of Drying Gas was 8 L/min at the temp of 325 °C; The flow rate of sheath gas temp was 12 L/min at the temp of 350 °C; The nebulization gas was set at 40 psi, The Nozzle Voltage was set at 0 V, Fragmentor was set at 150 V and the Vcap at 4000 V. For the negative ionization mode, the conditions were the same except the Nozzle Voltage set to 500 V. To ensure accuracy and reproducibility m/z (121.050873, 922.009798) in positive mode and (112.985587, 1033.988109) in negative mode were used as the lock mass. Data were collected in centroid mode.

### Data analysis

All of the MS data were stored as “.d” format and then converted into “.cef” format with the Profinder software (Agilent, Santa Clara, CA). Finally, the Mass Profiler Professional Software (MPP) was used to perform no-targeted metabolomics analysis. Only the compounds with a minimum absolute abundance of 2000 counts and more than 2 ions found were selected for the subsequent analysis. The peaks in the chromatograms of different samples were aligned using a retention time window of 0.3 min ± 0.2% and a mass window of 20 ppm ± 2.0 mD. Then, the peak intensity from different samples was normalized to the median of each group. The normalized data were processed by PCA using the MPP software to find all the significant compounds. These compounds were identified by searching in METLIN Database (Agilent) and comparing the accurate mass charge ratio with other databases, such as the HMDB and KEGG database.

### Statistical analysis

Data are represented as the mean ± SD. Statistical analysis of the results was performed using one-way ANOVA with Tukey’s correction for multiple comparisons. All of the data were analyzed using GraphPad Prism software (UK). A value of *P* < 0.05 was considered statistically significance.

According to methods from the literatures^59^, simple mathematical tools were used to normalize data from each individual raw behavioral data to the mean of the ND groups within each experimental cohort. Data from the OFT, TST and FST were then integrated into a single value, which were named emotionality z-scores. In addition, the intensity of differential compounds was extracted respectively and normalized named Z-intensity. The linear relationship between the emotionality Z-scores and Z-intensity was analyzed using the Pearson’s r after a Shapiro-Wilk normality test. *P* < 0.05 and | Pearson r | >0.5 was considered statistical correlated.
